# The Integrative Effects of Biochar and ZnO Nanoparticles for Enhancing Rice Productivity and Water Use Efficiency under Irrigation Deficit Conditions

**DOI:** 10.3390/plants11111416

**Published:** 2022-05-26

**Authors:** Omnia M. Elshayb, Abdelwahed M. Nada, Ahmed H. Sadek, Sameh H. Ismail, Ashwag Shami, Basmah M. Alharbi, Bushra Ahmed Alhammad, Mahmoud F. Seleiman

**Affiliations:** 1 Rice Research and Training Center, Field Crops Research Institute, Agricultural Research Center, Sakha 33717, Egypt; omniaelshayb3434@yahoo.com (O.M.E.); nadaabdelwahed456@gmail.com (A.M.N.); 2 Housing and Building National Research Center (HBRC), Sanitary and Environmental Engineering Research Institute, Giza 11511, Egypt; ahsadek@zewailcity.edu.eg; 3 Faculty of Nanotechnology for Postgraduate Studies, Sheikh Zayed Campus, Cairo University, Giza 12588, Egypt; drsameheltayer@yahoo.com; 4 Department of Biology, College of Sciences, Princess Nourah bint Abdulrahman University, P.O. Box 84428, Riyadh 11671, Saudi Arabia; Ayshami@pnu.edu.sa; 5 Department of Biology, Faculty of Science, University of Tabuk, Tabuk 71491, Saudi Arabia; b.alharbi@ut.edu.sa; 6 Biology Department, College of Science and Humanity Studies, Prince Sattam Bin Abdulaziz University, Al Kharj Box 292, Riyadh 11942, Saudi Arabia; b.alhammad@psau.edu.sa; 7 Plant Production Department, College of Food and Agriculture Sciences, King Saud University, P.O. Box 2460, Riyadh 11451, Saudi Arabia; 8 Department of Crop Sciences, Faculty of Agriculture, Menoufia University, Shibin El-Kom 32514, Egypt

**Keywords:** rice, deficit irrigation, biochar, zinc oxide NPs, productivity, water use efficiency

## Abstract

Water stress is considered one of the most environmental hazards that threaten agricultural productivity. Therefore, two field experiments were conducted to investigate the impact of biochar (6 t ha^−1^ as soil amendment), ZnO NPs (50 mg L^−1^ as foliar application), and their combination on growth, yield, and water use efficiency (WUE) of rice grown under four irrigation deficit treatments (i.e., irrigation every 3, 6, 9 and 12 d). The irrigation every 3 d was considered as the control in the current study. For this purpose, biochar was prepared through the pyrolysis of corn stalk and rice husk at 350 °C for 3 h, while sonochemical combined with the precipitation method was used to prepare zinc oxide nanoparticles (ZnO NPs) from zinc acetate. The morphological structures of the produced biochar and ZnO NPs were characterized using X-ray diffraction (XRD), N_2_ gas adsorption-desorption, scanning electron microscopy (SEM), and transmission electron microscopy (TEM). The results exhibited that the combination of biochar alongside ZnO NPs resulted in a positive significant effect on the physiological traits such as chlorophyll content, relative water content, plant height, and leaf area index as well as yield-associated components (i.e., number of panicles m^−2^, number of filled grain per panicle, 1000-grain weight), and biological and grain yield ha^−1^ when rice plants were irrigated every 9 days without a significant difference with those obtained from the control treatment (irrigation every 3 d). In conclusion, the combination of biochar and ZnO NPs could be recommended as an optimal approach to maximize both grain yield ha^−1^ and WUE of rice.

## 1. Introduction

Rice (*Oryza sativa* L.) as a cereal crop is an important food source for the most of the world’s population [1]. According to the rapid increase in the population rate, an increase in world rice production from 1 to 1.2% has to be achieved annually [2]. However, around 24–30% of the total global water is consumed by rice due to the high water needs [3]. In this case, water availability is a global issue for achieving its arability and security either by producing new cultivars that are tolerant to water stress or by using different treatments to mitigate the adverse effects of water stress on plants. Because of its growth nature (semi-aquatic), a malfunctioning rice physiological pathway has occurred under water deficit stress. Accordingly, this can impact negatively the growth, physiological and crop productivity traits grown under severe water stress [4]. With a long exposure stress period, the plants turn to generate active oxygen species (AOS). Singlet oxygen, superoxide anion radical, hydrogen peroxide, and hydroxyl radicals, as known as ^1^O_2,_ O_2,_ H_2_O_2,_ and OH^−^ respectively, are the main four types of AOS that can oxidize cell biological molecules (i.e., protein, chlorophyll, carbohydrate, and nucleic acid). Recently, the term of irrigation deficit (DI) has been recommended as an accurate technique that works on scheduling the irrigation inputs by supplying a precise and specific amount of water to the cultivated soil. This irrigation scenario is intended to minimize water requirement inputs and maximize water use efficacy, especially in the case of water poverty [5,6].

Scientists work hard to formulate and innovate modern techniques for improving water retention capacity and water use efficacy to secure rice cultivation and its productivity, especially in those areas affected by water shortages. Amongst different techniques, biochar utilization has wider benefits in the agriculture sector. The final product of organic biomass undergoes pyrolysis and limited oxygen supply resulting in producing a carbon-rich material known as biochar (BC). The physicochemical properties of BC (i.e., high surface area and reactivity, cation exchange capacity, and abundant functional groups) and economic availability make the biochar an attractive and efficient material for agriculturists’ goals [7,8]. Biochar applications have been proven benign and beneficial to soil in terms of improving both structure and porosity, increasing fertility, reducing water needs by enhancing water retention via enhancing moisture-holding capacity [9], and lessening soil evaporation [10], and increasing water productivity [11]. Also, it is a good activator to reinforce the availability of some nutrients in soil such as nitrogen [12], potassium, and phosphor [13] which make it benign supported to obtaining highly yield magnitude under harsh environments [14,15,16]. Moreover, BC contains silicon that can mitigate the negative effects of water stress [16]. Furthermore, pyrolysis of agricultural wastes and feedstocks such as rice husk or corn stalk is considered a sustainable soil organic amendment from the environmental point of view which can stimulate microbial activities in agricultural soils. On the other hand, BC utilization contributes to achieving environmentally-friendly agriculture by decreasing both nitrate leaching and nitrous oxide emission which directly dwindled the negative effect of climate change [11,12]. On the above basis, the utilization of rice husk and corn stalk’s biochar under deficit irrigation can be an innovative solution to sustain agricultural productivity for strategies crops such as rice in arid and semi-arid regions.

Zinc is a vital micronutrient for the healthy and vigorous growth of plants [17]. The positive effect of Zn functionalities has been concluded as an imperative activator or regulator of the various biochemical process in the plant system (auxin metabolism, chlorophyll formation, nucleotides outputting, and enzyme activation) and physiological process (carbohydrate, lipid, and protein synthesis, pollen formation, both DNA and RNA metabolism) as indicated by [18]. Owing to their elegant synthesis and smart delivery for agrochemicals, nano fertilizers offer novel nutrient systems in a new era. Zinc oxide nanoparticles (ZnO NPs) introduce unique forms of nano-sizing oxide, which are used to enhance crop growth but also, improve several nutrient uptakes [19,20]; mitigate the devastating effect of both droughts [20], salinity [20,21], and heavy metals [14,20], and as an efficient antimicrobial agent [20,22]. On that basis, the dual effect of biochar as amended soil application and zinc oxide nanoparticles as ameliorate exogenous application was applied in the current investigation to mitigate the negative effects of severe water deficient to optimize rice grain productivity.

Therefore, the current investigation objective was to evaluate the impact of rice husk’s and corn stalk’s biochar as a soil amendment and ZnO NPs as exogenous application either individuals or in combination on rice growth, physiological, yields, and water use efficiency traits under various water-deficit treatments. The hypotheses of the current investigation were that using rice husk’s and corn stalk’s biochar as soil amendment in combination with nano zinc oxide as exogenous application could mitigate the adverse effects of water-deficient when rice is grown and improve the water use efficiency.

## 2. Materials and Methods

### 2.1. Experimental Location and Soil Sampling

Over two successive seasons (2019 and 2020), field experiments were set up at the experimental farm of Sakha, Kafrelsheikh, Egypt (northwest delta) at 31°50′ latitude and 30°56′ longitude. The monthly temperature averages and relative humidity during both planting seasons are introduced in Table 1.

Bulk soil samples were collected diagonally and randomly from topsoil (depth at 0–30 cm) to analyze the physical and chemical (physiochemical) characteristics of the experimental soil samples. Soil analysis has been done according to the methods described by [23] as shown in Table 2.

### 2.2. Preparation and Characterization of Biochar

Biochar was processed via slow pyrolysis of corn stalk and rice husk (by ratio 1:1) at 350 °C for 3 h ceaseless under specific conditions of oxygen limitation to get product with low pH because the pH of the soil in the current study is alkaline (pH 8.35–8.45; Table 2). The corn stalk and rice husk were selected to be used for biochar production due to their abundance in the Egyptian environment as organic feedstocks. The obtained raw material was well ground and then it passed through a 2.0 mm mesh to remove large particles. Briefly, the initial physicochemical properties of biochar are analyzed and presented in Table 3.

### 2.3. Preparation of ZnO NPs

**Figure 1:**
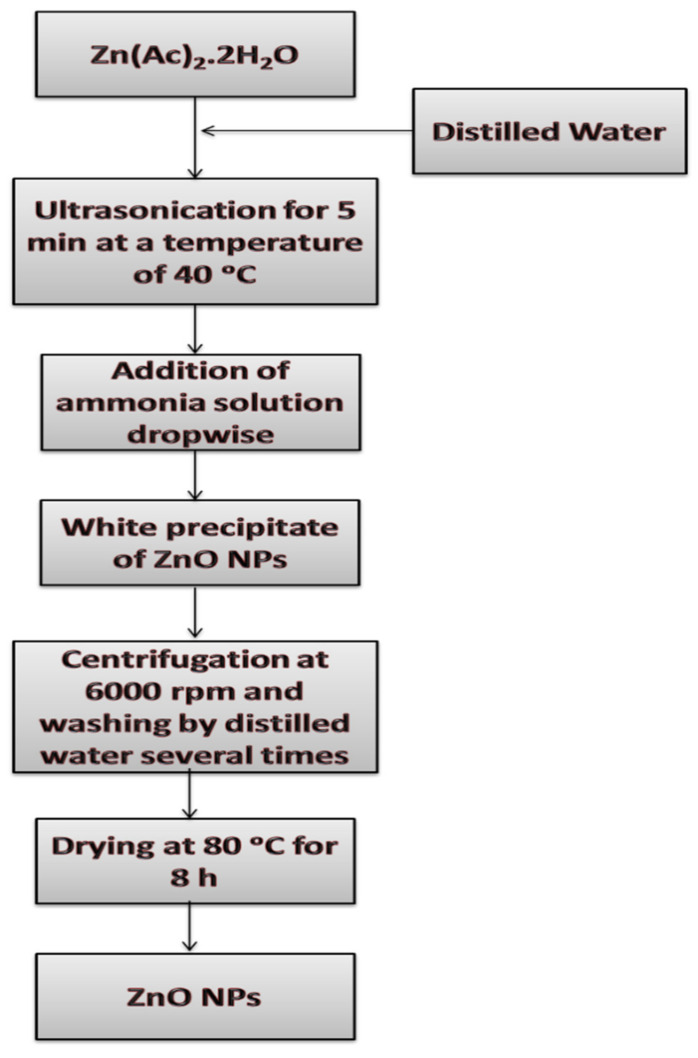
Flowchart for the preparation of ZnO NPs.

Incorporation between the chemical precipitation method and ultrasound irradiation was selected to prepare ZnO NPs. Firstly, an appropriate amount of zinc acetate dihydrate (Zn(CH_3_COO)_2_·2H_2_O, Loba Chemie, Mumbai, India) was dissolved in distilled water to obtain 0.1 M of Zn^2+^ ions solution. Subsequently, the Zn^2+^ solution was introduced to ultrasonic bath (Hielscher UP400S, Germany) at (400 W, 24 kHz, with an amplitude of 79% and a cycle of 0.76) for 5 min at a temperature of 40 °C. In the next step, an ammonia solution of 30–33% (NH_4_OH, Advent chembio, Mumbai, India) was added dropwise to the Zn^2+^ solution. In the meantime, a slight white precipitate appears with each drop of ammonia solution. With time, the solution turns off white as the reaction proceed. Afterward, the prepared ZnO NPs were separated from the supernatant by centrifugation at 6000 rpm and then were washed with distilled water many times. Finally, the collected ZnO NPs were dried in a thermal oven at 80 °C for 8 h. The synthesis process can be described as shown in Figure 1.

(1) $$\mathrm{Zn}{\left({\mathrm{CH}}_{3}\mathrm{COO}\right)}_{2}\cdot 2H_{2}O\;\overset{\mathrm{Ultrasonication}}{\to }\;{\mathrm{Zn}}^{2+}+2{\mathrm{CH}}_{3}{\mathrm{COO}}^{-}+2H_{2}O\;$$

(2) $${\mathrm{Zn}}^{2+}+2{\mathrm{NH}}_{4}\mathrm{OH}\;\overset{\mathrm{pH}\geq 9.5}{\to }\;\mathrm{Zn}{\left(\mathrm{OH}\right)}_{2}+2{\mathrm{NH}}_{4}^{+}\;$$

(3) $$\mathrm{Zn}{\left(\mathrm{OH}\right)}_{2}\;\overset{400W,24\;\mathrm{kHz}}{\to }\;\mathrm{ZnO}+H_{2}O\;$$

### 2.4. Characterization

The prepared ZnO NPs were investigated for chemical composition and crystalline phase using an X-ray diffractometer (XRD, D8-Discover, Bruker, Billerica, MA, USA) at a current of 30 mA and voltage of 20 kV. N_2_ adsorption at 77 K was used to assess the surface area of the ZnO NPs sample using the Brunauer-Emmett-Teller (BET) technique and a surface area analyzer (Autosorb-1, Quantachrome Instruments, Boynton Beach, FL, USA). The prepared sample was degassed at 200 °C for 9 h before examination. The total surface area was calculated using the multipoint BET equation. Field Emission Scanning Electron Microscope (SEM, JSM-6701F Plus, JEOL, Nieuw-Vennep, The Netherlands) was used to examine the surface morphology of all of the prepared ZnO NPs samples. Meanwhile, a Transmission Electron Microscope (JEOL, TEM-2100, Peabody, MA, USA) was utilized to examine the particle shape of the prepared ZnO NPs. The analysis was conducted by dropping a drop of the sample on carbon-coated grids and left to dry before investigation.

### 2.5. Experimental Details and Treatments

The prior cultivated crop was wheat during both seasons of the study. Pre-germinated healthy rice seeds (*Oryza sativa* L. CV. Giza 179) at the rate of 120 kg/ha were soaked in abundant water for 24 h and further incubated for another 48 h to enhance germination. Germinated seeds were broadcasted in the nursery on 12 May and 15 May during the 2019 and 2020 seasons, respectively. Three seedlings at 25 days old were transplanted in the permanent field’s experimental plots at a 20 × 20 cm distance between hills and rows in size plots of 15 m^2^ (5 m × 3 m). Weeds were controlled chemically using the commercial herbicide of Saturn [S-(4-chlorophenol methyl) diethyl carbamthioate] at the rate of 5 L/ha at 5 days after transplant. Nitrogen in the form of urea (46% N) at the rate of 165 kg N ha^−1^ was applied as recommended in two batches, 66.67% as a basal application and 33.33% at panicle initiation. Calcium superphosphate (15% P_2_O_5_) and potassium sulfate (48% K_2_O) were applied at a recommended dose at a rate of 37 kg P_2_O_5_/ha and 50 kg K_2_O/ha, respectively.

The permanent cultivable location of the experiment was well plowed (plowing two perpendicular times) and leveled. The experiment was laid out as a split-plot design with four replicates. The main plots were devoted to the four irrigation deficit treatments; irrigation every 3 (control), 6, 9, and 12 days which are denoted as ID3, ID6, ID9, and ID12, respectively. Whilst, the subplots were occupied by the four treatments of control, exogenous application of ZnO NPs at 50 mg/L, biochar (BC) at 0.6 kg/m^2^ (6 t/ha) as soil amendment, and their combination between the exogenous application of ZnO NPs and biochar application (BC + ZnO NPs). The BC was distributed and mixed with soil (depth, 0–20 cm) homogeneously in treated plots. However, treatments of ZnO NPs had been applied at two physiological stages of rice (i.e., mid–tillering and panicle initiation).

### 2.6. Measurements

#### 2.6.1. Physiological Traits

Chlorophyll content in flag leaves was measured using a SPAD meter device (SPAD-502, Minolta Sensing Ltd., Japan). Leaf area index (LAI) was estimated in the heading stage as described by [24] according to the following equivalent:

LAI = Leaf area (cm^2^) of specific plants ÷ ground area (cm^2^) covered by plants (4)

Relative water content (RWC) was estimated according to [25] as given in the following formula below:

RWC (%) = [(FW − DW) ÷ (TW − DW)] × 100(5)

FW: the weight of healthy and fresh leaves.
TW: the watery saturated weight after rehydrating leaves in distilled water for 24 h.
DW: the dry weight of water-saturated leaves after drying procedure at 80 °C for 24 h in an oven.

#### 2.6.2. Yield-Associated Components and Grain Yield per ha

Reproductive tillers of five hills were counted from the midmost of each plot at maturity to estimate the number of panicles/m^2^. Ten panicles were randomly collected to determine the total filled grains number/panicle, unfilled grains number/panicle, and 1000-grain weight (g). Biological yield and grain yields per ha were measured from an area of 12 m^2^ (3 × 4 m) which was harvested from the inner of each plot to avoid the border effects. Grain yield was adjusted to 14% moisture content. Harvest index (HI) was calculated as the ratio of economic yield (equivalent total grain weight) to biological yield (the subtotal of grain and straw weight) according to [26].

#### 2.6.3. Water Relations

Experiment irrigation has been conducted using calibrated water meter with a water pump to estimate the amount of applied water according to each treatment. Water use efficacy (WUE) was calculated by the following formula:

WUE = Grain yield outcome (kg/ha) ÷ the aggregate water applied (m^3^) (6)

### 2.7. Statistical Analysis

The obtained data from the effects of biochar, ZnO NPs and their combinations treatments on growth, physiology and productivity of rice grown under irrigation deficit were subjected to analysis of variance according to [27]. The means of treatments were compared using Duncan’s Multiple Range Test [28]. All statistical analyses were performed using PASW statistics 21.0 (IBM Inc., Chicago, IL, USA).

## 3. Results

### 3.1. Characterization of Biochar

**Figure 2:**
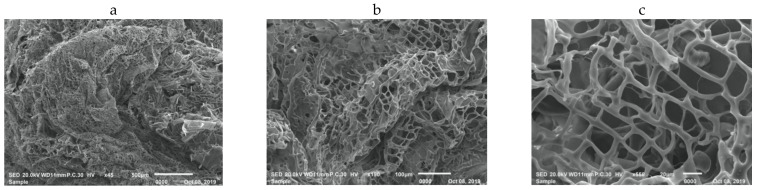
SEM images of the as-synthesized biochar at low and high magnifications (**a**) 500 µm; show the homogenous surface of biochar, (**b**) 100 µm; confirms the good porosity of prepared biochar (**c**) 20 µm; exhibit that the biochar formed in hollow network structures, respectively.

SEM was used to clarify the surface morphology of the as-synthesized biochar, since such parameters are important for biochar characteristics that can affect the soil and plants in next sections. Taking into account, the scanning was carried out at three different magnifications to observe all presented features formed with biochar. It can be observed from Figure 2a that the biochar materials exhibit flake-like morphology, where size varied in micrometer ranges. By increasing the magnification of the SEM image to 100 µm and 20 µm as shown in Figure 2b,c respectively, it can be seen the formation of hollow structures which are in µm sizes.

### 3.2. Characterization of Synthesized ZnO NPs

**Figure 3:**
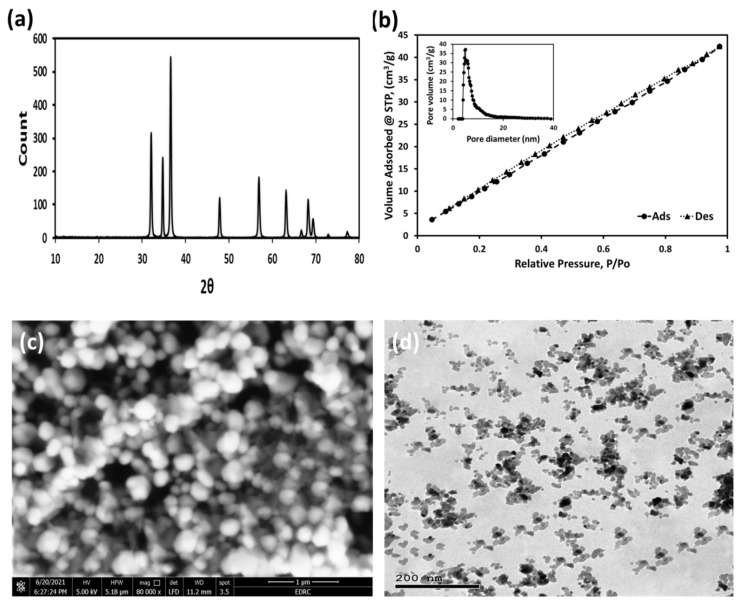
XRD pattern (**a**), BET of N_2_ adsorption-desorption isotherm showing IV type and the measured pore diameters/volumes curve (inset) (**b**), SEM image (**c**), and TEM image depicting the particles with sizes ≤ 50 nm (**d**) of the synthesized ZnO NPs.

X-ray diffraction was used to determine the structural characteristics of ZnO NPs. Figure 3a shows the XRD pattern of synthesized ZnO NPs. The identical eight major diffraction peaks of ZnO NPs confirmed the successful formation of ZnO NPs. The diffraction peaks were found at 31.93° (100), 34.62° (002), 36.44° (101), 47.81° (102), 56.89° (210), 63.24° (103), 68.34° (212), and 69.47° (201). The structure of the formed ZnO NPs is wurtzite (hexagonal phase, space group P63mc). The hexagonal phase of ZnO NPs given in the JCPDS card (No. COD 2300113, a = 3.2342 Å, c = 5.1772 Å) correspond to all of the obtained diffraction peaks. The results show that the ZnO NPs formed entirely in good pure phases. The XRD pattern showed no diffraction peaks related to any impurities, therefore, confirming the excellent purity of synthesized ZnO NPs. The crystalline size of the ZnO NPs found to be 48.70 nm as calculated using the Scherrer formula [29]:

(7) $$D=\frac{k\lambda }{\beta_{\mathrm{hkl}}\cos\theta }$$

where D is the crystalline size of ZnO NPs, λ is the X-ray wavelength, θ is the Bragg diffraction angle, β is the full-width at the half maximum of the diffraction peak corresponding to plane (101), and k is the Scherrer constant which is 0.9.

Figure 3b shows the nitrogen adsorption/desorption isotherms and pore size distributions of the synthesized ZnO NPs. The isotherms are classified as type IV and the hysteresis loops as type H3 indicating that the ZnO NPs have a mesoporous structure. The capillary condensation that occurs in mesopores causes a type IV isotherm. According to Figure 3b, the ZnO samples prepared in the way described above have a significant specific surface area of 54.26 m^2^/g. As shown in the inset figure, the pore volume and mean pore diameter were 0.063 cm^3^/g and 2.3 nm, respectively. SEM imaging was used to analyze the shape of ZnO NPs. As shown in Figure 3c, the resulting forms of ZnO NPs are very clear and homogeneous as evidenced by the SEM image. According to the SEM photograph, the average size of the majority of nanoparticles is in the nano domain. According to the TEM image, the ZnO NPs seem generally spherical and the average particles size is ≤50 nm (Figure 3d). The image also revealed the uniform size and consistent shape distribution of the obtained ZnO NPs. Furthermore, as seen in Figure 3d, there are several single ZnO crystallites morphologies with a good degree of crystallinity, besides portions of the ZnO NPs appearing together in the form of aggregates.

### 3.3. Changes in Physiological Traits

The physiological traits are very important to be measured to show the negative impacts of water stress or the mitigation role of BC and ZnO NPs in plants grown under irrigation deficit in further improving water productivity and drought adaptation is infinite, since such traits are considered the real indicator or the plant health and productivity in different stages. Data presented in Table 4 show that a significant impact on chlorophyll content, leaf area index, and relative water content was linked with increasing irrigation deficit (i.e., from ID3 to ID12 treatment). The findings highlighted that ID12 recorded the highest decline in the chlorophyll content by 43.4 and 42.7%, LAI by 33.8 and 34.0%, and RWC by 16.7 and 14.9% compared to ID3 in the first and second seasons, respectively.

In addition, there was not a statistical difference between ID3 and ID6 in all physiological studied traits in both cultivated seasons except for the RWC characteristic where a moral statistic difference between them occurred in the 2019 season. As for the applications of ZnO NPs, BC, and BC + ZnO NPs, it is clear that these treatments have positively improved on all physiological traits studied (Table 4). The best response was recorded under BC + ZnO NPs treatment which increases chlorophyll content by 14.5 and 13.6 %, leaf area index by 20.0 and 21.7 %, and relative water content by 12.7 and 14.9 % compared to control treatment in both 2019 and 2020 seasons, respectively. From the obvious results, the interactive treatment of ID3× BC + ZnO NPs generated the best findings of SPAD values in comparison to the other interactive treatments (Table 5). However, the lowest value of the interactive treatments has been noted when plants grown under ID12 treatment with no additional materials (control) in both cultivated seasons.

### 3.4. Changes in Yield-Associated Components and Grain Yield

#### 3.4.1. Plant Height, Number of Panicles/m^2^, Number of Filled and Unfilled Grains/Panicles

Although, irrigation deficit stress can strain growth of rice plants at any phenological stage, all these negative effects can culminate in final crop grain yield and its components. As compared to no irrigation deficit ID3, ID12 significantly decreased plant height (by 21.8 and 24.2%); both number of panicles/m^2^ (by 44.1 and 43.3%), and filled per panicle (by 43.2 and 44.1%) in 1^st^ and 2^nd^ seasons, respectively (Table 6). Inversely, the empty grains have witnessed a great increase in their number under ID12 in both cultivated seasons. ID3 plants were rendered statistically identical with ID6 but ID9 gave a quadratic response about above- mentioned traits in both seasons. The applications of ZnO NPs, BC, and BC + ZnO NPs are more successful in improving plant height, both number of panicles/m^2^ and filled per panicle, and diminishing unfilled grains number compared to control treatment in both seasons. However, the combination of BC + ZnO NPs resulted in an increment in plant height by 10.8 and 12.1%, number of panicles/m^2^ by 18.6 and 19.0%, number of filled/panicle by 22.8 and 24.0%, and a reduction in the sterility percentage by 33.5 and 31.8% as compared to control treatment in both experimental seasons, respectively.

As shown in Table 7, the interactive treatments provided the most outstanding number of panicles/m^2^ underlying both ID3 × BC + ZnO NPs and ID6 × BC + ZnO NPs treatments without significant difference among them in the 2019 and 2020 seasons. Whilst, the least panicles number per m^2^ is located under the interactive treatments of ID12× control in both seasons. Concerning the number of filled grains/panicle, the same trend was displayed in 2019, whereas the combination of ID3 × BC + ZnO NPs and ID6 × BC + ZnO NPs rendered the choicest number of filled grains/panicle in comparison to all interactive treatments (Table 8). A meaningful increase was witnessed under the combination of ID9 × BC + ZnO NPs regarding both numbers of panicles/m^2^ and filled grains/panicle in 1st season which recorded conformity statistics with ID3 × BC + ZnO NPs and ID6 × BC + ZnO NPs.

#### 3.4.2. Thousand-Grain Weight, Biological Yield, Grain Yield, and Harvest Index

The traits such thousand–grain weight, biological yield, grain yield, and harvest index are a result of several traits/mechanisms throughout the plant life. Such traits ultimately have an influence on irrigation deficit susceptibility index and consequently are considered as integral traits. Data presented in Table 9 show the different effects of irrigation deficit on 1000-grain weight, biological yield, grain yield and harvest index which gave the highest values when the irrigation was applied each 3 days (control treatment) or each 6 days without significant differences. In the 1st and 2nd seasons, results of variance analysis provide a significant reduction in 1000-grain weight by 7.9 and 7.7%, biological yield by 27.8 and 26.7%, grain yield by 36.8 and 37.2%, respectively, when plants were subjected to ID12 in comparison to ID3 that was considered control treatment (Table 9). However, the irrigated plants every 9 days (ID9) occupied the intermediate rank in the above-mentioned traits followed by the treatments of ID3 and ID6, which were statistically similar in both cultivated seasons. The given data indicated that a meaningful improvement has been obtained progressively by the application of ZnO NPs, BC, and their combination, i.e., BC + ZnO NPs (Table 9). The highest increase and improvement were obtained when soil and plants were treated with the combination of BC + ZnO NPs, which contributed to the increment in 1000-grain weight by 5.5 and 6.1%, biological yield by 15.1 and 16.2% and grain yield by 26.1 and 28.7% in both cultivated seasons compared to untreated soil and plants, respectively.

As for the interactive treatments, the weight of 1000 grains, biological yields, as well as grain yield was affected significantly by various treatments in both seasons (Table 9, Table 10 and Table 11). In each season, the lowest values of the previous parameters have been illuminated under the interactive treatment of ID12 × control. The highest value of 1000 grain was noticed under ID3 × BC + ZnO NPs treatment followed by ID3 × BC + ZnO NPs with no significant difference between them in the 2019 season. However, the treatment of ID9 × BC + ZnO NPs gave statistical identically with ID3 × BC + ZnO NPs and ID6 × BC + ZnO NPs in the 2020 seasons. The same trend was observed regarding both biological and grain yields in the 1st and 2nd seasons (Table 11 and Table 12). The highest outcome values of both biological and grain yields were rendered under the interactive treatment of ID3 × BC + ZnO NPs with a statistically match with ID6 × BC + ZnO NPs and ID9 × BC + ZnO NPs in each season.

#### 3.4.3. Water Relations

Data listed in Table 13 refer that different ID applications had a considerable variation in aggregate water applied (m^3^/ha), yield reduction (%), water saved (%), and water use efficacy (kg/m^3^) in 2019 and 2020 seasons. It is obvious that ID3 represented the highest value of aggregate water applied (m^3^/ha), whilst the least amount of water applied was recorded under ID12 in both cropping seasons. At the same time, ID3 treatment occupied the lowest percentage of both water saving and grain yield reduction. However, ID12 treatment rendered the highest percentage of both water saving and grain yield reduction in 1st and 2nd seasons. Interestingly, in each season, prolonged irrigation intervals until 9 days (ID9) had the maximum value of water use efficacy. The irrigation treatment every 6 days (ID6) gave the second-best efficacy of water use in both rice seasons. Therefore, irrigation treatment every 9 days could be an ideal application in this study.

**Figure 4:**
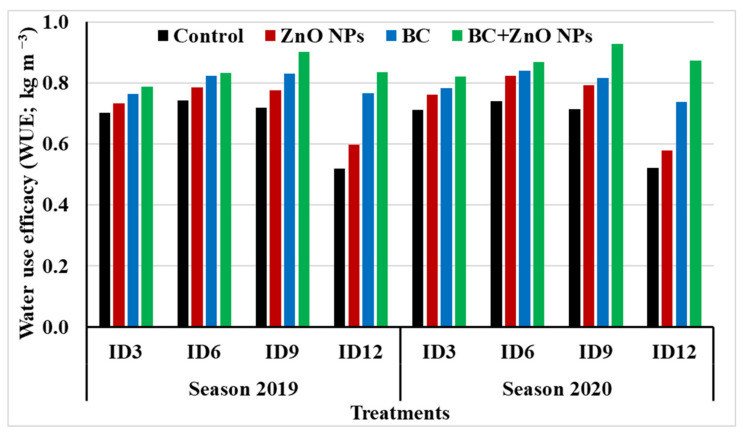
Interaction effects between irrigation deficit and applications of BC, ZnO NPs, and their combination treatments on water use efficacy (WUE) during 2019 and 2020 seasons. See Table 5 for abbreviations.

Concerning the combination effect of irrigation deficit and applications of BC, ZnO NPs, and BC + ZnO NPs treatments on water use efficacy, appraisal results pointed to the superiority of ID9 × BC + ZnO NPs in giving the most efficient use of water in both seasons followed by ID6 × BC + ZnO NPs in 2019 and 2020 seasons (Figure 4). However, the lowest value of water use efficacy was witnessed underlying the application of ID12 without any treatment addition in both seasons.

## 4. Discussion

### 4.1. The Effect of Irrigation Deficit on Growth, Yield Associated Component, Grain Yield and Water Relation

Benign growth traits are conclusive proof of a healthy plant environment. Additionally, the plant’s chlorophyll content is evidence of photosynthetic efficacy because it acts as a transfer and harvester of light energy [30]. Indeed, prolonged ID conditions are associated negatively with dire consequences in plant growth environments such as disabilities in nutrient absorption, absence of soil moisture that grossly affected the photosynthetic process, and relative water content [31,32,33]. Hence, the deterioration in physiological attributes under ID9 and ID12 (Table 4) is a logical result because the plant water needs are less than their required rates. In the water deficit case, inhibition in some key enzyme activities (such as 1,5 biophosphate carboxylase/oxygenase and rubisco activase) via increasing active oxygen species induced photosynthetic constraining during the reproductive phase [34]. Hence, probably, carbon fluxion to several reproductive organs is reduced consequently increasing pollen sterility, triggering ovary abortion, and impaired grains outcome [35]. Overall, ID stressors chain plant population/area, number of bearing tillers, panicle peduncle elongation that affected negatively on panicle traits (filled grains number, both panicle, and grain weights), and biomass production. Herein, extending irrigation up to 12 days caused the highest impairment in yield associated components except for the empty grain value which extremely increase (Table 6 and Table 9). Presumably, the negative impact of ID condition is due to the inadequate stored assimilates and their translocation to developed organs which are reflected ultimately in grain yield output [36]. In this context, these results are in line with those indicated by [37] who recorded a high decline in grain yield when irrigated treatment was at 9 and 12 days (25.7, 32.8% and 23.8, and 36.2%, respectively, in both seasons) compared with control treatment.

Our findings deduced that the rice crop does not need large amounts of water to produce a high yield outcome if it has adequate and suitable water addition. Therefore, there wasn’t a significant difference between ID3 and ID6 concerning final grain output (Table 9). Probably, these results owing to wet and dry cycles which allow the exchange of gases between cultivated soil and the outside atmosphere [38]. Yet another possibility was indicated by [37] who pointed to a benign increase in NH^+^-N, NO_3_-N, and K nutrient concentrations in the soil when irrigation treatment was every 6 and 9 days. These results were well supported by the research studies conducted by [37,38,39].

### 4.2. The Effect of Combined BC and ZnO NPs Applications on Growth, Yield Associated Component, Grain Yield, and Water Relation

In the current study, the application of ZnO NPs as exogenous treatment improved the growth, physiological, yield and water use efficiency of rice grown under water stress treatments. Separately, the application of ZnO NPs protects the leaf surface from harmful sun rays by blocking UV radiation [40]. Probably the role of ZnO NPs under water stress can cause a significant increase in melatonin levels because it can act as a free radical scavenger in response to stressors effect which relieved drought-induced impairment of chloroplast and mitochondria [41]. Realistically, Zn presence operates on raising tryptophan levels in plant tissues. Tryptophan is closely linked with the biosynthesis of indol-3-acetic acid and melatonin. Consequently, ZnO NPs can enhance cell division, and biomass production, and delay the senescence of plant cells [42,43]. Exogenous application of ZnO NPs works on mitigated photosynthetic pigment deterioration and regulated stomal movement in plant leaves under ID conditions as indicated by Yu et al. [10] who reported that the osmotic adjustment action of ZnO NPs was noted in water-stressed corn plants when ZnO NPs was applied at 100 mg/L. On the other hand, Zn is a benign catalyst to stimulate the rubisco enzyme since it is responsible for the formation of about 30-50% of soluble protein in C_3_ plants and this is considered a good contributor for enhancing the efficacy of gas exchange underlying harsh conditions [42]. Furthermore, ZnO NPs application contributed to the enhancement of glycolysis metabolism, both sucrose and starch biosynthesis in water-stressed plant leaves [41].

In the current study, the unique synthesis of ZnO NPs (a uniform size with average particles ≤ 50 nm) via the chemical precipitation method (Figure 1) might result in an enhancement of the osmotic regulation via the alteration of the fluidity and the potential of endomembrane tissues, resulting in ameliorating both leaf water potential and relative water content (Table 3). Hence, ZnO NPs could act as ameliorator substances to mitigate the adverse effect of water stress on growth and yield productivity. Prior studies, Elamawi et al. [21] and Linglan et al. [44] reported a remarkable increase in the components and the final grain yield of saline-stress rice plants when sprayed with ZnO NPs in mid tillering and panicle initiation. Rizwan et al. [45] indicated that using an exogenous application of ZnO NPs at the rate of 100 mg/L can cause a significant increase in spikes and grains dry weight by 74 and 69% in wheat plants grown under Cd and oxidative stress compared with control, respectively. Besides, it restrains electrolyte leakage (i.e., the leak out of electrolytes outside the cell which affected negatively on cell membrane integrity) and protected leaf tissues from destructive drought situations [46,47]. In this context, Seleiman et al. [20] reported an enhancement for the productivity when ZnO NPs was applied on crops grown under water stress.

On the other hand, the controlled pyrolysis method of feedstocks material under limited oxygen conditions can lead to a sequester riches carbon materials (Figure 2). Biochar as soil amendment enhances water holding capacity and modified hydrological properties of BC-treated soils; consequently can improve the biochemical and physiological traits of plants [10,48,49,50]. This implies that enabling water availability in cultivated soil that helps in completing several physiological processes inside plant cells. Maybe for those reasons, growing plants under BC-treated plots were characterized by relatively improved physiological traits compared to other treatments (Table 3). Sattar et al. [49] reported that BC application resulted in a substantial increase in the chlorophyll content, RWC, both shoot, and root dry matter of maize grown under drought stress. Prendergast-Miller et al. [51] reported an increase in the barley root biomass by 70% in BC treated soil which was a corollary to the water and nutrients availability in rhizosphere zones in comparison to untreated soil. Furthermore, the addition of BC leads to improve antioxidant enzyme activity, stomatal conductance, water relations, several nutrient uptakes, and organic matter statues which may be a good environment for the growth of plants grown under water stress [15,16]. Yeboah et al. [52] recorded a significant and positive effect on biomass production of maize when the soil was treated with BC at the rate of 5 t/ha. It has been established that, using BC improved wheat biomass outcome under semiarid Mediterranean regions [53]. Also, Abd-Elhamed [54] concluded that the application of BC at the rate of 6 t/ha underling saline soil contributed actively to increase yield components and maximizing final grain yield compared to other soil amendments.

Biochar properties can improve soil fertility and moisture-holding capacity, which can result in an amending for water requirements with maintaining benign yield production. Moreover, the sole application of BC enhances the water holding capacity of cultivated soil reflected positively in producing better water availability and improving the efficacy of water use feature [8,10]. The beneficial effects of BC application in the current study may be due to the maintaining suitable soil moisture around the root zone which can support plant system against drought stress [55]. Haider et al. [56] reported that BC application greatly enhanced the transpiration rate, osmotic potential, and leaf RWC of drought-stressed maize in comparison with those grown in untreated soil. Owing to the BC benefits around the rice root zone, it’s possible that BC can contribute effectively to reduce the wilting points whilst it increases soil moisture constants [57]. Hence, probably, BC addition as soil amendment to the cultivated soil could impact significantly water use efficacy to a great extent.

As per our findings during the current investigation, the combined application of BC and ZnO NPs resulted in a significant improvement into the physiological, yield components, and grain quantity traits compared to the individual application of either BC or ZnO NPs. This can be possibly due to the accompaniment of BC as soil amendment and ZnO NPs as exogenous applications which can enhance different nutrients acquisition and water enabling in roots zone as well as the amelioration effects of ZnO NPs on plant growth grown under irrigation deficit conditions. On this basis, the combined effect of BC as soil amendment and ZnO-NPs as exogenous applications can positively improve water relations and enhance crop yield production under irrigation deficit treatments.

## 5. Conclusions

Water irrigation deficit has a cardinal action in determining the final grain production, which grossly affected plant physiological traits, yield constituents, and consequently grain yield productivity. In our investigation, irrigation of rice plants each 3 or 6 days resulted in the highest values, without significant differences, of plant height, number of panicles m^−2^, number of filled grains per panicle, biological yield, grain yield and 1000-grain weight, while the lowest values of those traits were obtained when plants irrigated each 9 days. Under a severe water deficit, the influence would be devastating on the plant without the interference of agronomic treatments that would alleviate this negative effect. Accordingly, biochar preparation through the pyrolysis of the corn stalk and the rice husk (wastes) showed flake-like morphology with varied sizes in micrometer ranges and the formation of hollow structures that are extended to micrometer sizes. In addition, the sonochemical precipitation of synthesized ZnO NPs gave homogeneous nanoparticles with a hexagonal phase and crystalline size of 48.70 nm, a specific surface area of 54.26 m^2^/g, pore-volume, and mean pore diameter of 0.063 cm^3^/g and 2.3 nm, respectively. Though, Amendment of paddy soils with BC which is carbon-rich material has been proposed as a safe approach to abate water deficit cases via enhanced water availability in root growth zone environments. On the other hand, the application of ZnO NPs is considered unique stimulants nutrients that mitigate the undesired influence of water stress to an appreciable extent. The application of BC alongside ZnO NPs foliar sprayed has a synergistic impact on the enhancement of the physiological traits and yield-related attributes, and water use efficacy compared with the sole application. In the paddy cultivation system, more applied research is needed to validate these obtained findings under diverse varieties, soil types, different stressors as well as various climatic conditions.

## Figures and Tables

**Table 1:** Average monthly temperature and relative humidity datasets during 2019 and 2020 seasons.

	Parameters	Minimum Temperature °C	Maximum Temperature °C	Relative Humidity (%)
Seasons				
1st season (2019)				
June		25.4	31.9	57.1
July		28.3	33.5	69.8
August		28.9	34.2	70.65
September		27.9	32.4	68.15
October		26.7	30.3	70.8
2nd season (2020)				
June		23.8	32.0	53.7
July		27.3	33.7	67.7
August		28.2	34.6	67.5
September		27.1	34.2	67.2
October		24.6	31.5	65.9

**Table 2:** The physicochemical properties of cultivated experimental soil.

Character	2019	2020
**Physical analysis**		
Texture	Clay	Clay
Sand (%)	13.4	16.3
Silt (%)	32.0	28.0
Clay (%)	54.6	55.70
		
**Chemical analysis**		
pH (1:2.5 soil extract)	8.35	8.45
EC (dSm^−1^)	1.90	2.30
Organic matter (%)	1.51	1.65
Available N (mg/L)	17.30	18.20
Available P (mg/L)	14.20	15.30
Available K (mg/L)	313.0	318.0
Available Zn (mg/L)	0.85	0.90
Available Mn (mg/L)	3.10	3.93
Available Fe (mg/L)	2.64	2.96

**Table 3:** The physicochemical properties of biochar.

Properties	Unit	BC Sample
**Physical analysis**		
Moisture content	g/kg	34.0
Water holding capacity	g/kg	952.0
Bulk density	g/cm^3^	0.2
		
**Chemical analysis**		
pH	-	7.40
EC	dSm^−1^	0.70
N	g/kg	24.10
P	g/kg	8.40
K	g/kg	13.81
CaCO_3_	%	1.50
Ash	%	7.10

**Table 4:** Chlorophyll content, leaf area index, and relative water content of rice as affected by irrigation deficit and applications of BC, ZnO NPs, and their combination during 2019 and 2020 seasons.

Treatments	Chlorophyll Content (SPAD Values)		Leaf Area Index (LAI)		Relative Water Content (%)		
	2019	2020	2019	2020	2019	2020	
Irrigation deficit							
ID3	42.71 a	43.69 a	6.85 a	7.03 a	93.54 a	93.94 a	
ID6	41.93 a	43.12 a	6.73 a	6.86 a	92.42 b	91.70 a	
ID9	37.22 b	38.45 b	5.38 b	5.51 b	85.88 c	86.42b	
ID12	24.14 c	25.00 c	4.53 c	4.64 c	77.93 d	79.88 c	
Significance	**	**	**	**	**	**	
Treatments of BC and ZnO NPs							
Control	33.80 c	35.02 d	5.34 d	5.46 c	82.14 d	82.00d	
ZnO NPs	36.01 b	36.93 c	5.72 c	5.79bc	85.96 c	87.42 c	
BC	37.47 a	38.51 b	6.02 b	6.10 b	89.03 b	90 37 b	
BC + ZnO NPs	38.72 a	39.79 a	6.41 a	6.65 a	92.64 a	94.24 a	
Significance	**	**	**	**	**	**	
Interaction/Significance	**	**	ns	ns	*	*	

*, ** = ** indicates *p* ≤ 0.05 and 0.01, respectively; ns= not significant. Different letters (a, b, c, d, etc) in each column indicated that the means of the treatments were statistically varied at *p* ≤ 0.05. ID3: irrigation every 3 days; ID6: irrigation every 6 days; ID9: irrigation every 9 days, and ID12: irrigation every 12 days. Control: without any material application, ZnO NPs: foliar application of ZnO NPs at 50 mg/L; BC: application of biochar; BC + ZnO NPs: a combination of BC and ZnO NPs foliar application.

**Table 5:** Interaction effects between irrigation deficit and applications of BC, ZnO NPs and their combination treatments on chlorophyll content during the 2019 and 2020 seasons.

Treatments	Irrigation Deficit							
	2019				2020			
	ID3	ID6	ID9	ID12	ID3	ID6	ID9	ID12
Control	40.11 cd	39.94 cd	32.77 f	21.70 h	41.32 cd	41.04 de	34.91 g	22.84 i
ZnO NPs	42.95 ab	41.51 b	36.06 e	22.67 h	43.62 ab	42.82 b	37.19 f	24.22 i
BC	43.64 a	42.82 ab	38.27 d	25.26 g	44.50 ab	44.20 ab	39.50 e	25.92 h
BC + ZnO NPs	44.14 a	43.51 ab	41.35 b	25.77 g	45.32 a	44.48 ab	42.71 b	27.11 h

ID3: irrigation every 3 days; ID6: irrigation every 6 days; ID9: irrigation every 9 days, and ID12: irrigation every 12 days. Control without any treatment application, ZnO NPs: foliar application of ZnO NPs at 50 mg/L; BC: application of biochar; BC + ZnO NPs: a combination of BC and ZnO NPs foliar application. Different letters (a, b, c, d, etc.) in each column indicated that the means of the treatments were statistically varied at *p* ≤ 0.05.

**Table 6:** Plant height, number of panicles/m^2^, number of filled grains/panicles, and number of unfilled grains/panicles of rice as affected by irrigation deficit and applications of BC, ZnO NPs, and their combination treatments during 2019 and 2020 seasons.

Treatments	Plant Height (cm)		Number of Panicles/m^2^		Number of Filled Grains/Panicles		Number of Unfilled Grains/Panicles	
	2019	2020	2019	2020	2019	2020	2019	2020
Irrigation deficit								
ID3	101.2 a	103.3 a	556.6 a	574.4 a	128.3 a	132.0 a	6.38 c	5.69 c
ID6	99.4 a	101.1 a	543.9 a	555.8 a	125.6 a	128.5 a	6.75 c	6.02 c
ID9	90.5 b	91.0 b	427.5 b	445.1 b	108.2 b	112.2 b	13.50 b	12.63 b
ID12	79.1 c	78.2 c	311.9 c	325.6 c	72.9 c	73.8 c	21.80 a	20.91 a
Significance	**	**	**	**	**	**	**	**
Treatments of BC and ZnO NPs								
Control	87.6 c	87.8 d	422.0 d	432.5 d	97.8 d	99.3 d	14.45 a	13.58 a
ZnO NPs	91.8 b	92.3 c	443.8 c	459.8 c	103.3 c	107.0 c	13.28 b	12.30 b
BC	93.7 b	95.0 b	476.4 b	490.6 b	112.8 b	117.3 b	11.10 c	10.22 c
BC + ZnO NPs	97.1 a	98.5 a	500.8 a	514.6 a	120.1 a	123.1 a	9.60 d	9.26 d
Significance	**	**	**	**	**	**	**	**
Interaction effect	ns	ns	**	**	**	ns	ns	ns

** = *p* ≤ 0.01; ns = not significant. See Table 4 for other abbreviations. Different letters (a, b, c, d, etc.) in each column indicated that the means of the treatments were statistically varied at *p* ≤ 0.05.

**Table 7:** Interaction effects between irrigation deficit and applications of BC, ZnO NPs, and their combination treatments on the number of panicles/m^2^ during 2019 and 2020 seasons.

Treatments	Irrigation Deficit							
	2019				2020			
	ID3	ID6	ID9	ID12	ID3	ID6	ID9	ID12
Control	536.2 a	502.2 ab	358.2 de	291.2 e	544.4 ab	510.4 c	376.8 ef	299.0 g
ZnO NPs	553.1 a	532.1 a	390.0 cd	300.1 e	569.3 ab	553.4 ab	410.5 de	311.6 fg
BC	556.9 a	553.4 a	446.9 bc	325.2 de	580.6 ab	572.3 ab	466.0 cd	334.5 fg
BC + ZnO NPs	580 3 a	588.2 ab	514.9 ab	343.1 de	589.7 a	584.9 a	530.2 bc	356.6 ef

See Table 5 for abbreviations. Different letters (a, b, c, d, etc.) in each column indicated that the means of the treatments were statistically varied at *p* ≤ 0.05.

**Table 8:** Interaction effects between irrigation deficit and applications of BC, ZnO NPs, and their combination treatments on the number of filled grains/panicles during the 2019 season.

Treatments	Irrigation Deficit			
	2019			
	ID3	ID6	ID9	ID12
Control	119.1 cd	113.3 d	94.4 e	65.5 g
ZnO NPs	124.9 ab	122.4 bc	99.0 e	66.1 g
BC	132.3 ab	131.4 ab	115.2 cd	73.5 fg
BC + ZnO NPs	137.1 a	135.6 a	124.4 ab	82.6 f

See Table 5 for abbreviations. Different letters (a, b, c, d, etc.) in each column indicated that the means of the treatments were statistically varied at *p* ≤ 0.05.

**Table 9:** Thousand-grain weight, biological yield, grain yield, and harvest index of rice as affected by irrigation deficit and applications of BC, ZnO NPs, and their combination treatments during 2019 and 2020 seasons.

Treatments	1000-Grain Weight (g)		Biological Yield (t/ha)		Grain Yield (t/ha)		Harvest Index (HI)		
	2019	2020	2019	2020	2019	2020	2019	2020	
Irrigation deficit									
ID3	27.05 a	27.11 a	24.10 a	24.45 a	10.56 a	10.84 a	44.08 a	44.25 a	
ID6	26.93 a	27.00 a	23.95 a	24.23 a	10.42a	10.70 a	43.93 a	44.11 a	
ID9	26.16 b	26.28 b	21.95 b	22.37 b	8.90 b	9.05 b	41.87 b	42.03 b	
ID12	24.92 c	25.02 c	17.40 c	17.92 c	6.67 c	6.80 c	39.92 c	40.10 c	
Significance	**	**	**	**	**	**	**	**	
									
Treatments of BC and ZnO NPs									
Control	25.48 d	25.47 d	20.25 d	20.44 d	8.02 d	8.13 d	41.31 d	41.36 d	
ZnO NPs	26.17 c	26.20 c	21.34 c	21.70 c	8.78 c	9.00 c	42.06 c	42.17 c	
BC	26.60 b	26.64 b	22.61 b	23.01 b	8.57 b	9.80 b	42.84 b	43.00 b	
BC + ZnO NPs	26.88 a	27.03 a	23.32 a	23.76 a	10.11a	10.47 a	43.60 a	43.86 a	
Significance	**	**	**	**	**	**	**	**	
Interaction effect	**	**	**	**	**	**	ns	ns	

** = indicates *p* ≤ 0.01; ns = not significant. See Table 4 for other abbreviations. Different letters (a, b, c, d, etc.) in each column indicated that the means of the treatments were statistically varied at *p* ≤ 0.05.

**Table 10:** Interaction effects between irrigation deficit and applications of BC, ZnO NPs, and their combination treatments on 1000-grain weight during 2019 and 2020 seasons.

Treatments	Irrigation Deficit							
	2019				2020			
	ID3	ID6	ID9	ID12	ID3	ID6	ID9	ID12
Control	26.25 d	26.13 de	25.52 fg	24.21 h	26.20 de	26.11 de	26.60 fg	24.10 i
ZnO NPs	26.97 b	26.62 cd	25.98 ef	25.00 g	27.01 ab	26.60 bc	26.15 de	24.96 h
BC	27.35 ab	27.40 ab	26.21 de	25.25 g	27.53 a	27.49 a	26.37 cd	25.26 gh
BC + ZnO NPs	27.60 a	27.57 a	26.93 b	25.41 g	27.72 a	27.66 a	27.00 ab	25.74 ef

See Table 5 for abbreviations. Different letters (a, b, c, d, etc.) in each column indicated that the means of the treatments were statistically varied at *p* ≤ 0.05.

**Table 11:** Interaction effects between irrigation deficit and applications of BC, ZnO NPs, and their combination treatments on the biological yield during the 2019 and 2020 seasons.

Treatments	Irrigation Deficit							
	2019				2020			
	ID3	ID6	ID9	ID12	ID3	ID6	ID9	ID12
Control	23.34 ab	22.95 ab	19.54 c	15.20 e	23.47 bc	23.15 cd	19.81 e	15.75 f
ZnO NPs	23.79 a	23.48 ab	21.45 b	16.63 de	23.94 ab	23.81 ab	21.85 d	17.00 f
BC	24.61 a	24.43 a	23.05 ab	18.34 cd	24.85 ab	24.71 ab	23.45 bc	19.04 e
BC + ZnO NPs	25.04 a	24.93 a	23.86 a	19.46 c	25.53 a	25.26 ab	24.35 ab	19.84 e

See Table 5 for abbreviations. Different letters (a, b, c, d, etc.) in each column indicated that the means of the treatments were statistically varied at *p* ≤ 0.05.

**Table 12:** Interaction effects between irrigation deficit and applications of BC, ZnO NPs, and their combination treatments on grain yield during 2019 and 2020 seasons.

Treatments	Irrigation Deficit							
	2019				2020			
	ID3	ID6	ID9	ID12	ID3	ID6	ID9	ID12
Control	9.91 ab	9.73 b	7.96 ef	5.10 g	10.02 b	9.66 bc	7.80 e	5.13 f
ZnO NPs	10.35 ab	10.29 ab	8.59 cd	5.85 g	10.73 ab	10.76 ab	8.64 cd	5.66 f
BC	10.80 ab	10.76 ab	9.19 bc	7.53 f	11.05 a	10.95 ab	9.39 bc	7.82 de
BC + ZnO NPs	11.14 a	10.90 a	10.02 ab	8.20 ef	11.57 a	11.34 a	10.18 ab	8.54 cd

See Table 5 for abbreviations. Different letters (a, b, c, d, etc.) in each column indicated that the means of the treatments were statistically varied at *p* ≤ 0.05.

**Table 13:** Effects of irrigation deficit (ID) on aggregate water applied (m^3^/ha), yield reduction (%), water saved (%), and water use efficacy (kg/m^3^) during 2019 and 2020 seasons.

Seasons	Deficit Irrigation	Aggregate Water Applied (m^3^/ha)	Water Saved (%)	Grain Yield Reduction (%)	Water Use Efficiency (kg m^3^)
2019	ID3	14,102	-	-	0.748
	ID6	13,061	7.38	1.32	0.797
	ID9	11,065	21.53	15.70	0.807
	ID12	9797	30.52	36.83	0.680
2020	ID3	14,078	-	-	0.768
	ID6	13,030	7.44	1.29	0.821
	ID9	10,902	22.56	16.51	0.830
	ID12	9750	30.74	37.17	0.698

## Data Availability

All data are presented within the article.
